# Ischemic treatment of the duodenal windsock web using the ultra-thin endoscope, with modified detachable snares

**DOI:** 10.1055/a-2589-1411

**Published:** 2025-05-22

**Authors:** Kunihiko Oguro, Shogo Noda, Tomoko Tamaru, Hirotsugu Sakamoto, Tomonori Yano, Hironori Yamamoto

**Affiliations:** 1 12838 Department of Medicine, Division of Gastroenterology, Jichi Medical University, Shimotsuke, Japan; 2 3158 Division of Gastroenterology, University of Alberta, Edmonton, Canada; 3 38067 Department of Gastroenterology, Fukuoka Tokushukai Hospital, Kasuga, Japan; 4 12838 Department of Endoscopic Research and International Education Funded by Fujifilm Medical Co., Jichi Medical University, Shimotsuke, Japan

A duodenal web, one of the causes of congenital duodenal obstruction, is a rare congenital anomaly 1 . Patients with a complete-type duodenal web exhibit duodenal obstruction early in infancy, whereas those with a fenestrated type may be diagnosed with obstruction in adulthood 2 . In addition, peristalsis and gravity of food can cause the web to balloon distally, taking on the form of a windsock 3 . This forms a duodenal intraluminal diverticulum.

Endoscopic treatment of the duodenal web has become feasible in recent years; however, recurrence and adverse events, including bleeding, are concerning 1 3 4 .

A 27-year-old woman had experienced occasional vomiting attacks after eating since childhood. She was suspected to have an intraluminal duodenal diverticulum on computed tomography ( Fig. 1 ) and was diagnosed with a duodenal windsock web on esophagogastroduodenoscopy ( Fig. 2 **a,b** ).

**Fig. 1:**
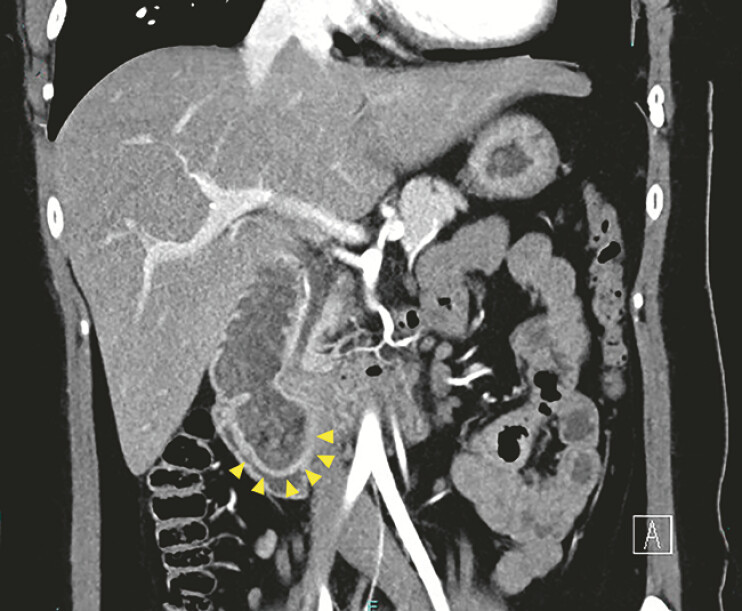
Computed tomography revealed a diverticulum in the second portion of the duodenum.

**Fig. 2:**
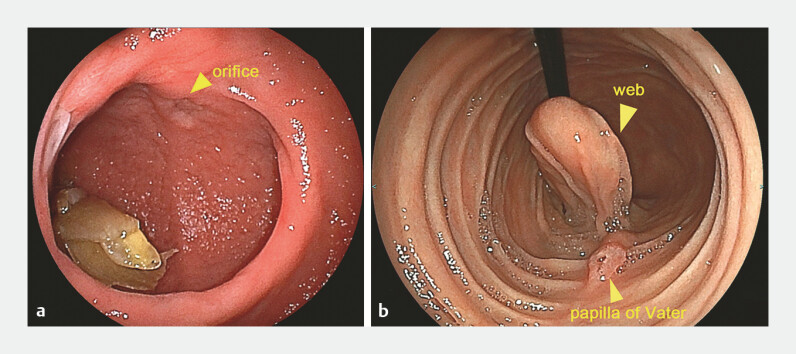
Endoscopic findings. **a** EGD from the oral side reveals a duodenal diverticulum and orifice similar to a pinhole. The orifice is located at the entrance of the true lumen. **b** EGD from the anal side reveals a web structuring the diverticulum. The papilla of Vater is found near the web. Abbreviation: EGD, esophagogastroduodenoscopy.

An ultra-thin endoscope (EG-530NW, Fujifilm) was passed through the narrow entrance of the true lumen ( Video 1 ). After confirming the location of the duodenal papilla in the reflex position, we tightened the neck of the web from the anal side using a detachable snare (HX-400U-30, Olympus) with the sheath removed to induce the web ischemia 5 . Two days later, the residual mucosa of the web near the necrotic area was tightened using a detachable snare. The following day, the web became entirely necrotic. We cut and removed the snares, made a new entrance to the lumen by penetrating the necrotic mucosa using forceps and scope, and then detained the clips at the side of the entrance. The patient’s abdominal bloating improved postoperatively. The new entrance was kept open, and the contrast medium was passed through the lumen without delay. The patient had no adverse events or recurrences. In conclusion, ischemic treatment in the reflex position is safe for duodenal webs.

Endoscopy_UCTN_Code_TTT_1AO_2AG_3AB

Case presentation of ischemic treatment for the duodenal windsock web.Video 1

## Notes

Endoscopy E-Videos https://eref.thieme.de/e-videos
*E-Videos* is an open access online section of the journal *Endoscopy* , reporting on interesting cases and new techniques in gastroenterological endoscopy. All papers include a high-quality video and are published with a Creative Commons CC-BY license. Endoscopy E-Videos qualify for HINARI discounts and waivers and eligibility is automatically checked during the submission process. We grant 100% waivers to articles whose corresponding authors are based in Group A countries and 50% waivers to those who are based in Group B countries as classified by Research4Life (see: https://www.research4life.org/access/eligibility/ ). This section has its own submission website at https://mc.manuscriptcentral.com/e-videos .
